# Dual-Biometric Human Identification Using Radar Deep Transfer Learning

**DOI:** 10.3390/s22155782

**Published:** 2022-08-02

**Authors:** Ahmad Alkasimi, Tyler Shepard, Samuel Wagner, Stephen Pancrazio, Anh-Vu Pham, Christopher Gardner, Brad Funsten

**Affiliations:** 1 Department of Electrical and Computer Engineering, University of California, Davis, Davis, CA 95616, USA; tashepard@ucdavis.edu (T.S.); samwagner@ucdavis.edu (S.W.); sbpancrazio@ucdavis.edu (S.P.); ahpham@ucdavis.edu (A.-V.P.); 2 Lawrence Livermore National Laboratory, Livermore, CA 95616, USA; gardner36@llnl.gov (C.G.); funsten1@llnl.gov (B.F.)

**Keywords:** millimeter-wave radar, FMCW, micro-Doppler signatures, human identification, convolutional neural networks, transfer learning, non-destructive sensing, security

## Abstract

Accurate human identification using radar has a variety of potential applications, such as surveillance, access control and security checkpoints. Nevertheless, radar-based human identification has been limited to a few motion-based biometrics that are solely reliant on micro-Doppler signatures. This paper proposes for the first time the use of combined radar-based heart sound and gait signals as biometrics for human identification. The proposed methodology starts by converting the extracted biometric signatures collected from 18 subjects to images, and then an image augmentation technique is applied and the deep transfer learning is used to classify each subject. A validation accuracy of 58.7% and 96% is reported for the heart sound and gait biometrics, respectively. Next, the identification results of the two biometrics are combined using the joint probability mass function (PMF) method to report a 98% identification accuracy. To the best of our knowledge, this is the highest reported in the literature to date. Lastly, the trained networks are tested in an actual scenario while being used in an office access control platform to identify different human subjects. We report an accuracy of 76.25%.

## 1. Introduction

Increased demand for surveillance and security has accelerated research in human identification based on remote sensing technologies [1,2,3,4,5]. Radar, as a sensing modality, offers robustness and privacy compared to other technologies [6,7,8,9,10]. For example, in Refs. [7,9], the human identification is based on iris scanning, which is highly dependent on lighting conditions. Additionally, in Refs. [6,8], the identification is vision-based, which adds to the light dependency problem the aspect of subject-image privacy. Radar, on the other hand, is insensitive to both light and weather conditions and provides contactless sensing without compromising subject privacy.

Furthermore, motion-produced micro-Doppler signatures have been studied extensively to enable the use of radar sensors in the fields of human detection and activity recognition. As such, deep learning methods, including convolutional neural networks and recurrent neural networks, are being implemented for their capabilities to achieve state-of-the-art results by automatically learning features from the raw sensor data. For example, Ref. [11] extracts the micro-Doppler signatures of human aquatic activities for the purpose of classification using transfer machine learning, while Ref. [12] proposes a deep convolutional autoencoder to classify 3D micro-Doppler signatures extracted from various human activities. Moreover, Ref. [13] proposes a feature fusion algorithm based on a stacked autoencoder for synthetic aperture radar automatic target recognition. In Ref. [14], multiple micro-Doppler-based forms of echoes and a wide variety of deep learning structures are reported in a survey of the deep-learning-based human activity recognition. However, most of the published research so far has focused on the identification of humans using one single biometric signature in a segmented user-dependent approach.

Gait, which can be defined as the pattern of movement of the limbs during any type of motion over a solid substrate, has drawn much attention as a radar-based application [3,15]. Gait is considered a unique signature due to the physical and behavioral characteristics difference between individuals, which can be used as a biometric for human identification. As observed in the literature [16,17,18], micro-Doppler-based methods have been implemented using a variety of machine learning structures with the purpose of identifying human targets based on their gait signatures. The main challenges are to maximize the accuracy of identification, minimize the need for larger training datasets and minimize the limitations imposed on the implementation scenarios. Although satisfactory results have been achieved, previous literature is still fully dependent on the target motion.

Additionally, heart sounds, which can be defined as the noises generated by the beating heart and the resultant flow of blood through it, are introduced in the proposed system as a unique biometric identifier due to the differences of people’s physical and, more specifically, cardiovascular characteristics. In previous literature [2,19], the related yet distinguishable radar-based heartbeat signal is proposed as a biometric identifier. While both signals can be extracted from the chest displacement waveform, the main difference is that heart sounds are smaller in magnitude and higher in frequency. The main advantage from choosing the heart sound signal is that it involves more than three distinct sounds that are related to, and indicative of, the condition of the heart. Consequently, this provides more features that can be utilized in classification.

Further, Refs. [20,21] demonstrate the feasibility of radar-based heart sound detection, and Ref. [22] proposes the electronic-stethoscope-based heart sound as a biometric for human identification. To the best of our knowledge, the use of the radar-based heart sound was not proposed in the literature to date as a biometric for human identification. While this work and Refs. [23,24] share the same methodology of extracting the target displacement by unwrapping the phase over the chirp time, Ref. [23] utilizes the extracted displacement waveform to detect the micron-level vibrations of reflective objects through a lossy material. Alternatively, Ref. [24] utilizes the extracted displacement waveform to sense anomalous drilling vibrations during vehicle transport on metallic containers. However, this work filters the extracted displacement waveform to detect the heart sound frequencies and generate time-frequency representations that can be further processed using image classification deep networks.

In this paper, a robust human identification system is proposed based on millimeter-wave frequency-modulated continuous wave (FMCW) radar that identifies human subjects based on gait and heart sound signatures with an accuracy of 98% using a fully autonomous signal processing pipeline. The combination of the two signatures improves the overall identification accuracy of the system. It also adds flexibility and robustness to the system as it enables the identification of human subjects in stationary scenarios where the motion-dependent gait-based identification is less advantageous. The system is also tested in an actual scenario to identify people belonging to an office. The purpose of this experiment is to evaluate the system performance as an identification platform. We believe that the developed methods can be useful for human identification in situations where a small to medium number of subjects follow a predictable route, such as in workplaces.

In summary, this paper makes the following contributions:

1. The use of the radar-based heart sound as a biometric identifier.
2. Combining two biometrics to improve the overall accuracy of the system and add flexibility.
3. Testing the system in an actual scenario to evaluate its performance as an identification platform.

## 2. Proposed Methodology

**Figure 1:**
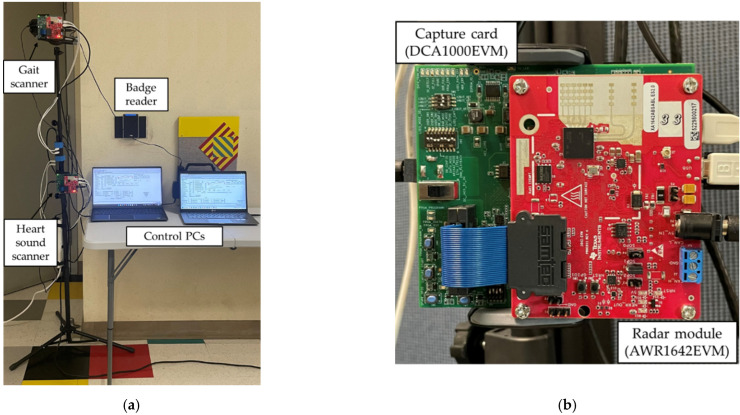
The data collection platform: (**a**) the complete setup incorporating two millimeter-wave radar sets and two laptops; (**b**) a radar set composed of radar module (front) and capture card (back).

To collect the data, the platform shown in Figure 1a was used. Two control laptops operate the two millimeter-wave radar sets using MATLAB and mmWave Studio software. Each of the two radar sets, as depicted in Figure 1b, is composed of a radar module, TI AWR1642EVM, and a capture card, TI DCA1000EVM. The mmWave Studio software is used to set the radar configuration parameters, trigger each scan and transfer the raw data files to the output directory. This process can be automated and executed in MATLAB using the LUA shell in the mmWave Studio software.

To construct the training dataset, 18 volunteers were scanned to measure both the heart sound and gait biometrics. Each volunteer was scanned 100 times for each biometric alternately. The subjects walked towards the platform to gather the gait signature using the upper radar set at an average pace of 1.36 m/second. Upon completion of the first scan, we started another scan of the subject, standing still, to gather the heart sound signatures using the lower radar set. After that, the augmentation technique is applied, which increased the number of samples by a factor of eight. This led to a total of 28,800 samples. The average height and weight of the volunteers are 171 cm and 76 kg, respectively. Additionally, their ages range from 22 to 50, with an average of 27 years. None of the participants reported any major health problems.

### 2.1. Heart Sound as a Biometric Identifier

In this section, we elaborate upon the scan parameters and signal pre-processing techniques used to generate the heart sound images for network training, validation and the practical testing. The FMCW waveform is a linear ramped time-frequency signal over the chirp ramp time, $T_{R}$, to cover chirp bandwidth, Δf.

In Equation (1), the transmitted FMCW signal ($S_{\mathrm{TX}}$) is defined where α is the chirp ramp rate, given as $\alpha =\Delta f/T_{R}$, and $f_{c}$ is the center frequency:

(1) STX(t)=Re{ ATX e j(2πfct+παt2)}

The reflected signal is then down-converted through mixing it with the transmitted signal, yielding an intermediate-frequency (IF) signal that is a function of the target reflectivity and echo time delay (τ). The down-converted IF signal is then passed through a low-pass filter. At this point, the IF signal for any given channel is:

(2) $$S_{\mathrm{IF}}\left(t,\tau \right){=\mathrm{LPF}\{\;S}_{\mathrm{TX}}\left(t\right){\hat{S}}_{\mathrm{TX}}\left(t\;-\tau \right)\}=\frac{1}{2}A_{TX}A_{RX}\mathrm{rect}\left(\frac{t}{T_{\mathrm{CRI}}}\right)e^{\;j\left({2\pi \alpha \tau t+2\pi f}_{c}\tau -{\;\pi \alpha \tau }^{2}\right)}$$

where $S_{\mathrm{TX}}$ is the transmitted signal, ${\hat{S}}_{\mathrm{TX}}\left(t\;-\tau \right)$ is the time delayed receive signal, $A_{\mathrm{TX}}$ is the transmitted waveform amplitude and $A_{RX}$ is the received signal amplitude, accounting for path losses. The IF signal is then digitized by an ADC at a fast-time sampling rate. Throughout a single pulse, the frequency of the down-converted signal contains the range by being linearly dependent on the echo time delay. There are two phase terms on the IF signal. The term that is quadratic in the echo time delay is typically ignored. The chirp is repeated at a slow-time chirp repetition interval (CRI), $T_{CRI}$.

**Figure 2:**
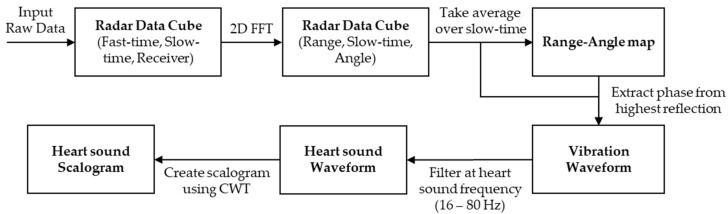
The signal processing pipeline for the heart sound biometric showing the major steps followed to extract the heart sound waveform and to convert it to an image (scalogram).

The goal of this processing pipeline shown in Figure 2 is to extract the subject’s heart sound vibration data from the radar returns. The first step in the signal-preprocessing chain is to determine the down-converted beat frequency. From Equation (2), the beat frequency can be determined by a Fourier transform over fast-time, referred to as a range FFT. The signal after the range FFT is now:

(3) $$S_{\mathrm{IF}}\left(f,\tau_{n}\right)=\frac{1}{2}A_{TX}A_{RX}*T_{\mathrm{CRI}}\frac{\sin\left(\pi \left(f-\alpha \tau \right)\right)}{\pi \left(f-\alpha \tau \right)}e^{{j2\pi f}_{c}\tau_{n}},n\in \;\left[1,N_{\mathrm{Pulses}}\right]$$

After the range FFT, the data are spatially focused by performing an angle FFT over the receive channels. To determine the subject’s location, the range and angle bin with the highest magnitude target return is chosen. The vibration data are linearly related to the phase of the range and angle FFT output when tracked at the target bin. For this step, we note that the IF phase differences mostly appear over the slow-time intervals. The next step is to extract the phase over the slow-time chirp repetitions and unwrap it to generate the vibration signal. At this point in the signal processing chain, the IF phase contains not only the heart sound vibration information but also the information from other vibrations unique to the target, such as breathing and small random movement. These interreferences are observed and expected since the targets are standing during the stationary heart sound measurements. They can make tiny random movements during the 10 s that are comparable in magnitude with the chest displacement caused by the heart sounds. Similarly, the breathing of the targets is not always limited to frequencies lower than 16 Hz. Subsequently, the waveform is filtered for the primary heart sound frequencies of 16–80 Hz using a brick-wall bandpass filter with a passband gain of 0 dB and a stopband gain of −60 dB, as in (4):

(4) $$S_{V}\left(n\right)=\mathrm{BPF}\;\left\{{\mathrm{unwrap}}_{\left(0,2\pi \right)}∠\left(S_{\mathrm{IF}}\left(f_{\mathrm{sel}},n\right)\right)\right\}\;$$

**Figure 3:**
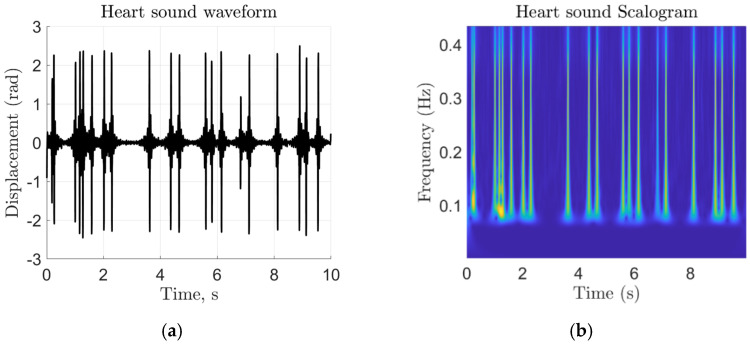
Heart sound signal representations as (**a**) a time-domain chest displacement waveform and (**b**) a time-frequency representation based on the absolute value of the CWT coefficients of (**a**), which is the final output corresponding to each scan.

At this stage, (4) represents the raw extracted target vibration signal. An example of the extracted vibration waveform can be seen in Figure 3a. Although the vibration signal is filtered for the most primary heart sound frequencies, other sources of 16–80 Hz vibrations will still be present. We rely upon the neural network to distinguish 16–80 Hz vibration features between subjects. To generate an image that can be used for transfer learning training and verification, a continuous wavelet transform (CWT) is applied to the heart sound signal to generate a time-frequency scalogram [25]. We extended the CWT method to PCG time-frequency image generation displayed in Figure 3b. A careful consideration is paid to the number of scans that were needed to train the network. By training with several different dataset sizes, we experimentally determined that one hundred scans per subject was sufficient to train the network. The trained network is then used to identify subjects in the second portion of the experiment, where we took ten new scans of each subject for the testing task.

In Table 1, we show the chirp parameters that were used based on the radar manufacturer recommendations for ultra-short-range scenes [26]. We further refined the recommended settings to ensure that the full bandwidth of the signal is captured. To achieve the Nyquist rate on the heart sound signal, a maximum $T_{CRI}$ of 8 ms is needed; we chose a $T_{CRI}$ of 5 ms. To increase the frequency domain resolution without unreasonably long scan times, we chose 2000 pulse repetitions for a single scan, yielding a scan duration of approximately 10 s. The fast-time chirp parameters were chosen to fill a 4 GHz pulse bandwidth over a 1 ms chirp duration. 

### 2.2. Gait as a Biometric Identifier

The second biometric feature used for the joint identification is gait. The task is to measure a walking subject using the mm-Wave radar and then extract the individual’s Doppler signature, which will be applied to transfer learning.

**Figure 4:**
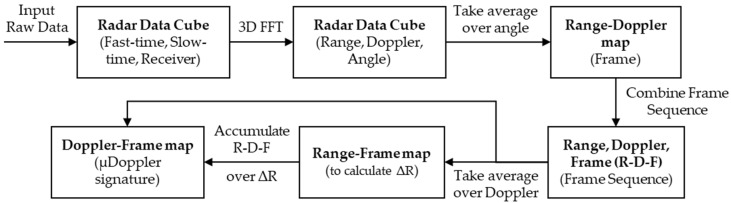
The signal pre-processing pipeline for the gait biometric used to extract the Doppler-frame heat map.

The gait preprocessing pipeline, as depicted in Figure 4, is used to generate a Doppler-frame image that could be used for training and identification purposes.

The proposed gait pre-processing pipeline starts with the radar data cube. We first perform a range FFT, similar to the first step of the heart sound signal processing. At this point in the pipeline, the signal is identical to that in Equation (3). In this case, however, the pulses are repeated over a frame at a much higher rate than that of the heart sound. From here, the velocity of the target is assumed constant over a single frame since the frame is relatively small (50 ms) compared to the velocity of the subject. We then gather sequential frames to allow for variation in velocity across the scan. Each frame is made up of $N_{\mathrm{Pulses}}$ number of repeated pulses.

(5) $$S_{\mathrm{IF}}\left(f,\tau_{n}\right)=\left|S_{\mathrm{IF}}\left(f,\tau_{n}\right)\right|{*e}^{\;j\;\frac{2\pi }{\lambda_{c}}\;(2r+2\left(n-1\right){\mathrm{vT}}_{\mathrm{CRI}})},n\in \left[1,N_{\mathrm{Pulses}/\mathrm{Frame}}\right]$$

As can be seen from Equation (5), the Doppler frequency due to target motion appears in the phase of the exponential, which can be extracted using a Fourier transform, henceforth referred to as the Doppler FFT. Finally, a Fourier transform is performed along the channel dimension of the data-cube and non-coherently integrated to obtain a range-Doppler plot for each frame. Equivalently, we can write the range-Doppler data as 3-D FFT, where the angle dimension is collapsed by summing over it.

(6) $$S_{m}\left(f_{IF},f_{d}\right)=\overset{N_{ch}}{\underset{k=1}{\sum }}{\left|{ℱ}_{3D}\left\{S_{IF}\left(n,p,k\right)\right\}\right|}^{2}$$

where $n\in \left[1,\;N_{Fast\;Time}\right]$ and $p\in \left[1,\;N_{Pulses/Frame}\right].$ To generate the Doppler-frame heatmap, we first slice each frame over the range dimension and look for the highest return. Then, a limited number of nearby range bins, ΔR, is selected and the range dimension is squeezed out by summing to generate the Doppler-frame heatmap:

(7) $$S\left(m,\;f_{d}\right)=\overset{R+\frac{\Delta R}{2}}{\underset{R-\frac{\Delta R}{2}}{\sum }}S_{m}\left(f_{IF},f_{d}\right)$$

**Figure 5:**
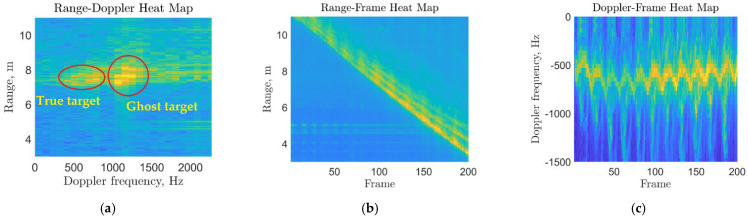
Gait signal processing outputs after the main processing blocks: (**a**) a sample for the range-Doppler map showing the targets’ ranges versus their frequency of motion in a single time frame; (**b**) a sample for the range-frame map showing target movement (range) towards the radar versus time frames; (**c**) a sample for the resulting Doppler-frame map showing the target’s body and limbs’ motion toward the radar.

In Figure 5, the samples for the outputs of the three main blocks (third, fifth and sixth) of the pipeline in Figure 4 are shown. Note that the range-frame heatmap clearly indicates the position of the target throughout the scan.

This position is tracked, and a limited number of adjacent bins are used to generate the Doppler-frame heatmap according to the processing pipeline.

In Table 2, we show the chirp parameters used to produce the optimal Doppler sampling for the gait biometric. Initial chirp parameters were based off the parameters of previous gait experiments [17]. These parameters are set to cover an unambiguous Doppler frequency of ±2 kHz or a radial target velocity of ±3.8 m/s. To cover the unambiguous Doppler spectrum, we set the chirp repetition interval and the chirp duration. For the fast-time parameters, we set a 4 GHz bandwidth over the 200 μs chirp ramp duration for the optimal target resolution in the range dimension.

### 2.3. Classification Using Deep Transfer Learning

While training the deep networks from scratch can achieve high classification accuracy [27], for smaller datasets (compared to ImageNet 1.5 million training samples), deep transfer learning shows superior performance as the former method may not realize the full potential of the deep network.

Transfer learning can be defined as the technique that transfers knowledge learned from one task to another related task that lacks sufficient training data. This technique improves the accuracy of classification given that the original and new datasets have some similarity [11]. While the actual implementation might differ from one application to another, a generalized procedure can be followed to apply transfer learning on a deep convolutional neural network (DCNN): choose a deep network that has been trained on a dataset similar to the targeted dataset, replace the output classification layer and fully connected layer with new layers that match your targeted output size and fine-tune the network parameters using the training dataset corresponding to your application.

**Figure 6:**
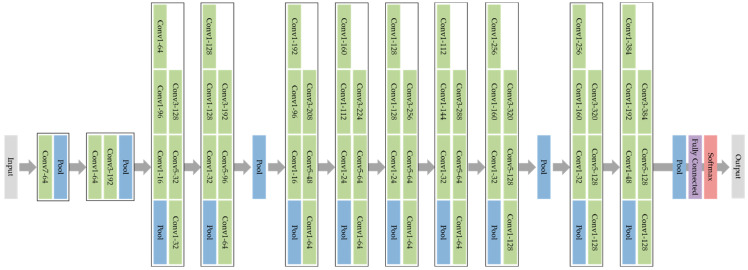
GoogLeNet architecture composed of a mixture of convolutional (green), pooling (blue) softmax (red) and fully connected (purple) layers.

To apply transfer learning, we use GoogLeNet, as depicted in Figure 6, which is a deep convolutional neural network that is 22 convolutional layers deep with about 6.8 million parameters. In MATLAB, GoogLeNet is pre-trained on either the ImageNet or Places365 datasets. We use the network trained on ImageNet, which classifies images into 1000 object categories, such as a mouse, a pencil, a keyboard and many other animals. The pretrained network has an image input size of 224-by-224, which can be matched to any input using the image resize function.

To train the network, the adaptive moment estimation optimizer is implemented using the hyper-parameters listed in Table 3. We then fine-tuned the pre-trained network parameters using the images corresponding to the heart sound and gait data.

The above implementation was carried out using the Deep Learning Toolbox in MATLAB 2021b and utilized using Intel Core i7-11800H processor and NVIDIA GeForce RTX 3050 TI GPU.

### 2.4. Image Augmentation Technique

**Figure 7:**
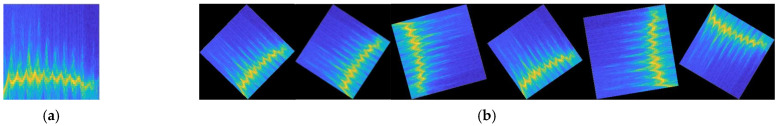
The image augmentation technique applied on the gait biometric: (**a**) a sample RGB image; (**b**) the resulting images after applying the random 2D affine function.

After training GoogLeNet, the resulting classification accuracy showed that the network performance is not optimal due to overfitting. Overfitting is defined as the phenomenon in which a network learns a function with a very high variance in a way that perfectly models the training data. This is common in many application domains that lack access to big data. To overcome this issue, we looked for a technique that provides a data-space solution to the applications of limited data. Subsequently, we compared the various techniques presented in Ref. [28] and found the best performance improvement in applying the rotation augmentation technique. To apply this technique, the random 2D affine function is used in MATLAB to randomly rotate the training images between −180 and 180 degrees, as depicted in Figure 7. 

Subsequently, eight new variations from each image are created, which increased the sample size by a factor of eight.

In Table 4, we show the results of training GoogLeNet on the heart sound data before and after applying the augmentation technique in which we see a significant improvement in the accuracy from 27.27% to 58.7%.

### 2.5. Joint Probability Mass Function (PMF) Method

After optimizing the classification results from each biometric independently, we combine the two prediction results using the joint probability mass function. To do so, we calculate the probability of the two classifiers predicting the same person assuming the two events are independent.

Therefore, we first define the joint probability mass function of two discrete random variables *X* and *Y* as follows:

(8) $$\begin{array}{c} P_{XY}\left(x,y\right)=P\left(X=x,\;Y=y\right)\\ \;\;\;=P\left(\left(X\cup Y\right)=\left(x,y\right)\right)\\ =P_{x}\left(x\right)⊗P_{y}\left(y\right) \end{array}$$

Next, we apply Equation (8) to the prediction scores array resulting from each classifier, which is a 1 × N array that specifies the prediction score for each of the N subjects, by extracting the diagonal elements of the Kronecker tensor product. We then normalize the resulting array to obtain a summation of one. The resulting 1 × N array shows the combined prediction scores from the two biometrics, which can be visualized as the probability of the detected target being any of the subjects scanned during the training process.

### 2.6. Practical Testing

In this section, the trained networks are deployed in an actual scenario and used in an access control platform. The purpose of this experiment is to evaluate the system performance in a practical implementation as an identification platform. To do that, the trained networks are implemented in classifying eight subjects out of the eighteen subjects who contributed to train the networks 4 months after the training data were collected. Ten samples are collected per biometric from each subject, which leads to a total number of 160 samples. After that, the samples are processed, and the results are compared to those obtained previously during the validation procedure. Feasibly, this platform could be used along with badge readers, which are commonly implemented to secure vital entrances. 

**Figure 8:**
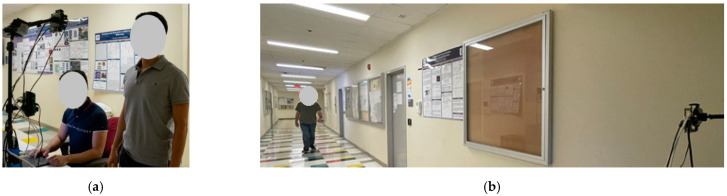
Data collection for the practical testing showing the subjects (**a**) standing for the heart sound scans and (**b**) walking for the gait scans.

In Figure 8, a sample of the data collection process is shown in which the subjects are asked to walk normally towards an office entrance while being scanned for the gait and heart sound biometrics sequentially. The total time needed to collect both biometrics is calculated to be 13 s.

**Figure 9:**
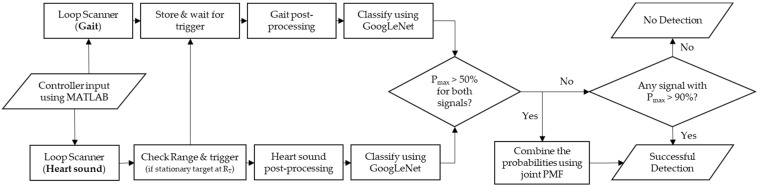
The signal processing pipeline used in the practical testing to classify the subjects and validate the data.

Figure 9 shows the autonomous signal processing pipeline designed to work with two millimeter-wave radar modules continuously.

The role of the signal processing pipeline is to run the classifiers each time the predefined conditions are met, decide whether the acquired scans are valid and provide an output that points to the identity of the detected target.

The process starts by an input from the operator using MATLAB by running the two scripts, one on each laptop, which will then start the loop scanners. The loop scanners will monitor the radar’s output folders continuously and move the raw data files to a shared folder that is accessible to the two laptops. Additionally, the gait scanner, also called secondary scanner, will store the last four files, each corresponding to a recording time of three seconds, which results in total storage time of 12 s.

Conversely, the heart sound scanner, also called the primary scanner, will store only one raw data file at a time corresponding to 10 s. During those loops, the primary scanner will check the scan files for a stationary target 50 cm away from the platform. If the target is detected, the primary scanner will issue a flag that will trigger the two classifiers. Then, the primary scanner will use the same scan to extract the heart sound biometric. Additionally, the secondary scanner will pick the least recent scan file corresponding to the time when the subject was walking towards the platform. Subsequently, the classifiers will process the data as explained in Section 2.1 and Section 2.2 to produce two images that can be fed to the trained neural networks. The neural networks will then classify the images independently and produce two outputs per each image. One output will return the name of the predicted class (i.e., name of the subject), while the other, in the form of a matrix, will return the prediction score, P, for each class. After that, the primary scanner will check the results to determine if the prediction scores for the predicted subjects are higher than 50%. If the condition is satisfied, the results are combined using the joint PMF method, as explained in Section 2.5, and the predicted subject is returned to the user with a successful detection mark. On the other hand, if any of the two predictions has a prediction score that is less than 50%, the primary scanner will check if either of the two predictions has a prediction score higher than 90%. If the result is true, it will return the predicted class from the biometric with the highest prediction score with a successful detection mark or, otherwise, return a no detection mark.

## 3. Experimental Results

In this section, we will show the results of training the neural networks for each of the biometrics and the combined validation accuracy of the proposed system. Then, we will go through the practical testing in which the trained networks are validated in an actual scenario to evaluate the system performance as an identification platform.

### 3.1. DCNN Training Results

**Figure 10:**
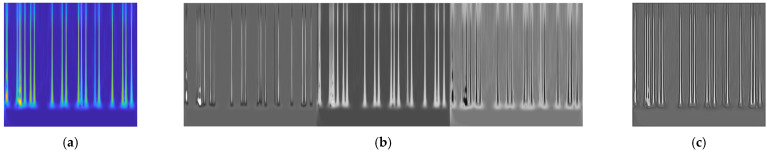
Features learned from the heart sound biometric: (**a**) a sample input image, which is an output of the heart sound signal processing pipeline; (**b**) the features extracted from the first convolutional layer; (**c**) the strongest feature in the third convolutional layer. White pixels represent strong positive activations and black pixels represent strong negative activations.

**Figure 11:**
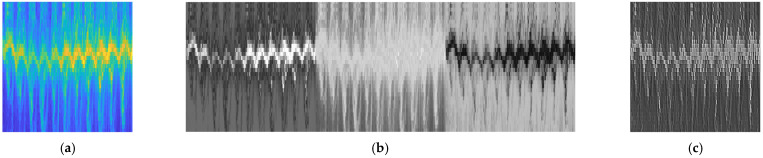
Features learned from the gait biometric: (**a**) a sample input image, which is an output of the gait signal processing pipeline; (**b**) the features extracted from the first convolutional layer; (**c**) the strongest feature in the third convolutional layer. White pixels represent strong positive activations and black pixels represent strong negative activations.

We trained the transfer learning network, GoogLeNet, using the parameters specified in Table 3 independently for each biometric. Then, we used 87% of the collected data to train each network and the remaining 13% to validate the training. In Figure 10 and Figure 11, we provide a visualization of sample input images corresponding to each biometric as it passes through the convolutional layers of the trained GoogLeNet network. 

Figure 10a and Figure 11a shows RGB images extracted from a random scan as an input, while Figure 10b and Figure 11b shows the three features learned from the first convolutional layer and Figure 10c and Figure 11c shows the strongest feature, among 192 features, learned from the third convolutional layer based on the activation strength.

Table 5 shows the confusion matrices resulting from the validation process, for a total number of subjects (N) of 18. The heart sound biometric achieves an average accuracy of 58.695%, with a maximum of 80% and a minimum of 30%. Comparing it with the random guess probability (RGP), which is given by: RGP = 1/N = 5.56%, we see that the heart sound average identification accuracy is ten times higher.

On the other hand, the average accuracy for the gait biometric is 96.256%, with a maximum of 100% and a minimum of 86%, which is significantly higher than the RGP and comparable to the best reported validation accuracies in literature for the gait-based classification [17].

Subsequently, the joint PMF method is applied to calculate the joint accuracy of the two networks for each subject. By comparing the combined identification accuracy of each subject to the single-biometric identification accuracies, we can see that the combination reduces the error rate in most cases, which is very useful in scenarios where one biometric is more capable of identifying the subject than the other. An example for such cases can be observed with subjects 6 and 13, where the gait identification accuracy is 88% and 86%, respectively. Comparing it with combined accuracy, we see that the heart sound increased the accuracy by 10% and 12%, respectively. However, on some rare occasions, the heart sound classifier predicts a subject incorrectly, with high prediction scores, which affects the results of the joint PMF method negatively. An example for such occasions can be observed with subject 9, where the accuracy of the combined biometrics is 6% less than that of the gait biometric.

Furthermore, in the first section of the table, we see that the heart sound classifier is producing significantly lower accuracies compared to the gait classifier. A major factor behind this degradation is that the filtered heart sound signals are incorporating vibrations generated from the small random movements of the target and from the chest displacement during breathing. While the heart sound signals that are unique to each target are seen by the radar and distinguished by the classifier, it is relatively small in magnitude and, therefore, affected by those interferences. Alternatively, for the gait biometric, we can relate the superior accuracies to the higher visibility of the targets’ micro-Doppler motions in the generated Doppler-frame heat maps. In part, we obtain very good results due to filtering the Doppler frequencies, which is applied to show only the negative domain that represents the target motion towards the radar. This yields to heat maps that are fully exploited in terms of the size of useful content inside each image.

In Table 6, the achieved validation accuracies are summarized and compared with the state-of-the-art radar-based human identification results published from 2016 to 2021. The accuracies are sorted based on the biometrics used in each work, the neural network implemented for classification and the number of participants involved in the experiments.

We see that most of the recent results reported in the literature for the radar-based identification are based on the gait biometric [4,15,16,17,29,30,31,32,33]. We can relate that to the superior accuracies achieved using the gait signature compared to other signatures, as in Ref. [2]. Additionally, a wide variety of deep learning structures are utilized for classification, with numbers of participants that range from 4 [33] to 100 [16]. While the comparison in this context is multi-dimensional, we compare our results to Ref. [17], in which transfer learning is utilized in classifying 20 subjects to achieve an accuracy of 96.70%. The gait-based classification reported in this work achieves an accuracy of 96.2%. We combine it with the heart sound biometric to demonstrate an accuracy of 98%, which is the highest radar-based human identification accuracy reported in literature so far. We note here that the time needed to train each network is 83 min, which leads to a total training time of two hours and 46 min.

### 3.2. Practical Testing

To further validate the reported training results, an experiment is conducted in which the trained networks are tested using radar scans that are captured a few months after the training scans to evaluate the system performance as an identification platform.

Table 7 shows the resulting confusion matrix for the combined predictions with 76.25% average identification accuracy. Compared to the validation result of 98%, we notice a drop of 20%, which can be justified by taking into consideration the variables incorporated into the walking pattern and the cardiovascular status of the subjects. Some of those variables are weight, clothing style, activity level, walking speed and new physical health changes. To compensate for the degeneration of accuracy observed on a few of the subjects, new training data could be collected to retrain the neural networks. This can be generalized to all future applications by asking the involved participants to take new training data every time they go through significant physical changes or in frequent time intervals so as to update the network with their new physical features.

## 4. Conclusions

In this paper, the feasibility of using radar gait and heart sound signatures was investigated to classify human subjects using deep transfer learning to provide a remote solution that does not suffer from privacy invasion for the problem of human identification. A dataset of 18 subjects was established in which the gait and heart sound measurements were collected using millimeter-wave radar. A preprocessing pipeline was then designed for each biometric in which RGB images were generated out of the micro-Doppler and vibration signatures to use the pretrained networks for image classification. Lastly, a validation experiment was designed in which the trained networks were implemented a few months after the collection of training data to evaluate the system performance as an identification platform. The contributions of this work can be summarized as follows: (i) the radar-based heart sound signal is implemented for the first time as a biometric for human identification; (ii) a platform that combines two radar-based biometrics is proposed to provide identification accuracy that outperforms the state-of-the-art results reported to date; (iii) the system is tested in an actual scenario to evaluate its performance as an identification platform.

Furthermore, as explained in the introduction, one of the promising applications for this study would be business environments where personnel identification is crucial for secure facilities. In addition to the advantage of privacy protection, the proposed system provides robustness to light and weather conditions, which makes it more suitable to be implemented in harsh outdoor environments where the vision-based systems might be less advantageous. Additionally, while this work involves a limited number of subjects, the proposed methodology can be applied to any number of subjects. The identification accuracy, however, is expected to be lower, as shown in Ref. [16], which is due to the higher complexity of the classification problem caused by the higher number of classes.

Notably, the gait measurements are not reaching 100% identification accuracy and can be improved by either further optimizing the neural networks’ parameters or by increasing the number of features extracted from the acquired data. The most significant limitations of this work are observed in the heart sound measurements. A potential future work direction might be in further investigating the acquisition process of the heart sound data and optimizing the filtering techniques in order to generate more distinguishable time-frequency representations. Additionally, a potential future research direction might be further investigation of human identification through walls, which expands the features of the radar-based systems. While the signal is expected to be weak and submerged in the clutter and noise, preliminary work results [34] show that the significant suppression of the primary wall reflections is possible using digital filters that enhance the target signal to clutter ratio.

Lastly, while the participants involved in this work are young and healthy, we expect a better performance on other mixtures of health statuses and ages that include wider ranges of variations. This is due to the anticipation of increased uniqueness in terms of the walking patterns and cardiovascular shape. Examples of such populations might be the residents of medical or senior living facilities.

## Figures and Tables

**Table 1:** Radar configuration parameters for heart sound measurements.

Parameter	Value
Carrier frequency (GHz)	77
Chirp duration (ms)	1
Frequency slope (MHz/μs)	4
ADC sampling rate (ksps)	512
Number of ADC samples per chirp	256
Number of chirps per frame	1
Chirp repetition interval (ms)	5
Number of chirps	2000
Tx/Rx channels	1/4

**Table 2:** Radar configuration parameters for gait measurements.

Parameter	Value
Carrier frequency (GHz)	77
Chirp duration (μs)	200
Frequency slope (MHz/μs)	20
ADC sampling rate (ksps)	3000
Number of ADC samples per chirp	256
Number of chirps per frame	100
Chirp Repetition Interval (ms)	30
Number of chirps	200
Tx/Rx channels	1/4

**Table 3:** Hyper-parameters used to train GoogLeNet.

Parameter	Value
Initial Learning Rate	0.0001
Gradient Decay Factor	0.95
Squared Gradient Decay Factor	0.99
Max Epochs	30
Mini-Batch Size	25

**Table 4:** The confusion matrices for the heart sound biometric with and without the image augmentation technique showing true classes (rows) versus predicted classes (columns) for 18 subjects (also known as classes). 1 indicates 100% of the samples in the corresponding class were predicted correctly, while 0 indicates no correct predictions. Classes are marked in green if the prediction accuracy is 90% or more.

Heart sound without the rotation technique		**1**	**2**	**3**	**4**	**5**	**6**	**7**	**8**	**9**	**10**	**11**	**12**	**13**	**14**	**15**	**16**	**17**	**18**
	1	0.18	0.27	0.00	0.18	0.00	0.00	0.00	0.09	0.00	0.00	0.00	0.00	0.00	0.00	0.18	0.00	0.00	0.09
	2	0.00	0.45	0.09	0.00	0.00	0.00	0.00	0.09	0.00	0.00	0.09	0.00	0.00	0.00	0.00	0.09	0.09	0.09
	3	0.00	0.00	0.27	0.27	0.00	0.09	0.00	0.09	0.00	0.00	0.00	0.00	0.09	0.00	0.00	0.00	0.18	0.00
	4	0.00	0.09	0.18	0.45	0.00	0.09	0.00	0.00	0.00	0.00	0.00	0.00	0.09	0.00	0.00	0.00	0.09	0.00
	5	0.00	0.00	0.09	0.09	0.18	0.00	0.00	0.36	0.09	0.09	0.00	0.00	0.00	0.00	0.00	0.00	0.00	0.09
	6	0.00	0.18	0.00	0.00	0.09	0.27	0.00	0.09	0.00	0.09	0.00	0.00	0.00	0.00	0.18	0.00	0.00	0.09
	7	0.00	0.09	0.00	0.00	0.00	0.00	0.09	0.09	0.00	0.00	0.09	0.00	0.00	0.00	0.00	0.18	0.00	0.45
	8	0.00	0.09	0.00	0.27	0.00	0.00	0.00	0.09	0.00	0.18	0.00	0.00	0.00	0.00	0.00	0.00	0.27	0.09
	9	0.00	0.00	0.00	0.00	0.00	0.00	0.00	0.09	0.27	0.64	0.00	0.00	0.00	0.00	0.00	0.00	0.00	0.00
	10	0.00	0.00	0.00	0.27	0.09	0.00	0.00	0.18	0.00	0.18	0.00	0.00	0.18	0.00	0.09	0.00	0.00	0.00
	11	0.00	0.27	0.00	0.09	0.00	0.09	0.00	0.00	0.00	0.00	0.00	0.00	0.00	0.00	0.27	0.00	0.09	0.18
	12	0.00	0.00	0.18	0.09	0.00	0.00	0.00	0.00	0.00	0.18	0.00	0.55	0.00	0.00	0.00	0.00	0.00	0.00
	13	0.00	0.00	0.00	0.18	0.00	0.00	0.00	0.00	0.00	0.00	0.00	0.00	0.73	0.00	0.09	0.00	0.00	0.00
	14	0.00	0.00	0.00	0.18	0.27	0.00	0.00	0.18	0.00	0.36	0.00	0.00	0.00	0.00	0.00	0.00	0.00	0.00
	15	0.00	0.36	0.00	0.00	0.00	0.00	0.00	0.00	0.00	0.00	0.00	0.00	0.00	0.00	0.45	0.09	0.00	0.09
	16	0.09	0.18	0.00	0.00	0.00	0.00	0.09	0.00	0.00	0.00	0.00	0.00	0.00	0.00	0.27	0.00	0.00	0.36
	17	0.00	0.36	0.00	0.09	0.00	0.00	0.00	0.00	0.00	0.00	0.00	0.00	0.09	0.00	0.18	0.00	0.27	0.00
	18	0.00	0.18	0.00	0.09	0.00	0.00	0.00	0.09	0.00	0.00	0.00	0.00	0.00	0.00	0.09	0.00	0.09	0.45
Heart sound with the rotation technique		**1**	**2**	**3**	**4**	**5**	**6**	**7**	**8**	**9**	**10**	**11**	**12**	**13**	**14**	**15**	**16**	**17**	**18**
	1	0.52	0.08	0.00	0.00	0.02	0.02	0.08	0.00	0.00	0.00	0.03	0.01	0.00	0.02	0.03	0.12	0.00	0.07
	2	0.02	0.63	0.05	0.01	0.00	0.01	0.02	0.02	0.00	0.00	0.02	0.00	0.00	0.01	0.05	0.02	0.07	0.05
	3	0.00	0.02	0.84	0.01	0.01	0.00	0.00	0.00	0.00	0.01	0.02	0.00	0.01	0.00	0.00	0.02	0.03	0.02
	4	0.00	0.04	0.17	0.55	0.00	0.00	0.00	0.02	0.00	0.03	0.03	0.01	0.01	0.03	0.04	0.01	0.02	0.01
	5	0.01	0.00	0.03	0.04	0.61	0.07	0.02	0.00	0.02	0.03	0.00	0.02	0.01	0.03	0.01	0.05	0.01	0.02
	6	0.00	0.03	0.03	0.02	0.00	0.63	0.05	0.03	0.01	0.07	0.02	0.00	0.00	0.00	0.00	0.02	0.00	0.08
	7	0.00	0.07	0.00	0.00	0.00	0.02	0.52	0.00	0.00	0.02	0.05	0.00	0.00	0.01	0.01	0.18	0.00	0.11
	8	0.01	0.05	0.03	0.07	0.05	0.03	0.03	0.33	0.07	0.04	0.01	0.03	0.05	0.00	0.04	0.01	0.08	0.05
	9	0.00	0.00	0.00	0.00	0.09	0.01	0.00	0.04	0.64	0.02	0.00	0.05	0.03	0.11	0.00	0.00	0.00	0.00
	10	0.02	0.02	0.05	0.01	0.03	0.07	0.01	0.03	0.11	0.38	0.00	0.05	0.03	0.08	0.02	0.04	0.01	0.02
	11	0.05	0.11	0.02	0.01	0.01	0.01	0.04	0.00	0.00	0.01	0.45	0.00	0.00	0.00	0.10	0.03	0.08	0.08
	12	0.01	0.02	0.01	0.01	0.01	0.00	0.00	0.00	0.04	0.00	0.00	0.87	0.00	0.02	0.00	0.00	0.00	0.00
	13	0.00	0.02	0.07	0.00	0.03	0.01	0.00	0.00	0.03	0.05	0.00	0.07	0.64	0.03	0.00	0.01	0.03	0.00
	14	0.02	0.01	0.02	0.01	0.13	0.01	0.00	0.02	0.07	0.05	0.00	0.08	0.05	0.49	0.00	0.01	0.00	0.02
	15	0.00	0.08	0.00	0.01	0.00	0.01	0.02	0.01	0.00	0.03	0.10	0.00	0.00	0.00	0.62	0.03	0.04	0.04
	16	0.00	0.05	0.00	0.00	0.01	0.05	0.02	0.01	0.00	0.00	0.03	0.01	0.00	0.00	0.10	0.65	0.01	0.04
	17	0.02	0.09	0.08	0.04	0.00	0.00	0.01	0.00	0.00	0.00	0.04	0.00	0.02	0.00	0.05	0.01	0.61	0.02
	18	0.08	0.07	0.01	0.01	0.02	0.10	0.01	0.00	0.00	0.01	0.02	0.00	0.00	0.01	0.05	0.01	0.01	0.59

**Table 5:** The confusion matrices for heart sound and gait biometrics showing true classes (rows) versus predicted classes (columns) for 18 subjects (also known as classes). 1 indicates 100% of the samples in the corresponding class were predicted correctly, while 0 indicates no correct predictions. Classes are marked in green if the prediction accuracy is 90% or more.

The heart sound biometric		**1**	**2**	**3**	**4**	**5**	**6**	**7**	**8**	**9**	**10**	**11**	**12**	**13**	**14**	**15**	**16**	**17**	**18**
	1	0.52	0.08	0.00	0.00	0.02	0.02	0.08	0.00	0.00	0.00	0.03	0.01	0.00	0.02	0.03	0.12	0.00	0.07
	2	0.02	0.63	0.05	0.01	0.00	0.01	0.02	0.02	0.00	0.00	0.02	0.00	0.00	0.01	0.05	0.02	0.07	0.05
	3	0.00	0.02	0.84	0.01	0.01	0.00	0.00	0.00	0.00	0.01	0.02	0.00	0.01	0.00	0.00	0.02	0.03	0.02
	4	0.00	0.04	0.17	0.55	0.00	0.00	0.00	0.02	0.00	0.03	0.03	0.01	0.01	0.03	0.04	0.01	0.02	0.01
	5	0.01	0.00	0.03	0.04	0.61	0.07	0.02	0.00	0.02	0.03	0.00	0.02	0.01	0.03	0.01	0.05	0.01	0.02
	6	0.00	0.03	0.03	0.02	0.00	0.63	0.05	0.03	0.01	0.07	0.02	0.00	0.00	0.00	0.00	0.02	0.00	0.08
	7	0.00	0.07	0.00	0.00	0.00	0.02	0.52	0.00	0.00	0.02	0.05	0.00	0.00	0.01	0.01	0.18	0.00	0.11
	8	0.01	0.05	0.03	0.07	0.05	0.03	0.03	0.33	0.07	0.04	0.01	0.03	0.05	0.00	0.04	0.01	0.08	0.05
	9	0.00	0.00	0.00	0.00	0.09	0.01	0.00	0.04	0.64	0.02	0.00	0.05	0.03	0.11	0.00	0.00	0.00	0.00
	10	0.02	0.02	0.05	0.01	0.03	0.07	0.01	0.03	0.11	0.38	0.00	0.05	0.03	0.08	0.02	0.04	0.01	0.02
	11	0.05	0.11	0.02	0.01	0.01	0.01	0.04	0.00	0.00	0.01	0.45	0.00	0.00	0.00	0.10	0.03	0.08	0.08
	12	0.01	0.02	0.01	0.01	0.01	0.00	0.00	0.00	0.04	0.00	0.00	0.87	0.00	0.02	0.00	0.00	0.00	0.00
	13	0.00	0.02	0.07	0.00	0.03	0.01	0.00	0.00	0.03	0.05	0.00	0.07	0.64	0.03	0.00	0.01	0.03	0.00
	14	0.02	0.01	0.02	0.01	0.13	0.01	0.00	0.02	0.07	0.05	0.00	0.08	0.05	0.49	0.00	0.01	0.00	0.02
	15	0.00	0.08	0.00	0.01	0.00	0.01	0.02	0.01	0.00	0.03	0.10	0.00	0.00	0.00	0.62	0.03	0.04	0.04
	16	0.00	0.05	0.00	0.00	0.01	0.05	0.02	0.01	0.00	0.00	0.03	0.01	0.00	0.00	0.10	0.65	0.01	0.04
	17	0.02	0.09	0.08	0.04	0.00	0.00	0.01	0.00	0.00	0.00	0.04	0.00	0.02	0.00	0.05	0.01	0.61	0.02
	18	0.08	0.07	0.01	0.01	0.02	0.10	0.01	0.00	0.00	0.01	0.02	0.00	0.00	0.01	0.05	0.01	0.01	0.59
The gait biometric		**1**	**2**	**3**	**4**	**5**	**6**	**7**	**8**	**9**	**10**	**11**	**12**	**13**	**14**	**15**	**16**	**17**	**18**
	1	1.00	0.00	0.00	0.00	0.00	0.00	0.00	0.00	0.00	0.00	0.00	0.00	0.00	0.00	0.00	0.00	0.00	0.00
	2	0.00	0.99	0.00	0.00	0.00	0.00	0.00	0.00	0.00	0.00	0.00	0.00	0.00	0.00	0.00	0.00	0.01	0.00
	3	0.00	0.00	0.97	0.00	0.03	0.00	0.00	0.00	0.00	0.00	0.00	0.00	0.00	0.00	0.00	0.00	0.00	0.00
	4	0.00	0.00	0.00	1.00	0.00	0.00	0.00	0.00	0.00	0.00	0.00	0.00	0.00	0.00	0.00	0.00	0.00	0.00
	5	0.00	0.00	0.00	0.00	1.00	0.00	0.00	0.00	0.00	0.00	0.00	0.00	0.00	0.00	0.00	0.00	0.00	0.00
	6	0.00	0.00	0.00	0.10	0.00	0.88	0.00	0.00	0.00	0.00	0.00	0.02	0.00	0.00	0.00	0.00	0.00	0.00
	7	0.00	0.00	0.00	0.00	0.00	0.00	1.00	0.00	0.00	0.00	0.00	0.00	0.00	0.00	0.00	0.00	0.00	0.00
	8	0.00	0.00	0.00	0.00	0.09	0.00	0.00	0.89	0.00	0.00	0.00	0.00	0.01	0.01	0.00	0.00	0.00	0.00
	9	0.00	0.00	0.00	0.00	0.00	0.00	0.00	0.00	0.99	0.00	0.00	0.01	0.00	0.00	0.00	0.00	0.00	0.00
	10	0.00	0.00	0.01	0.00	0.02	0.00	0.00	0.00	0.00	0.95	0.00	0.00	0.02	0.00	0.00	0.00	0.00	0.00
	11	0.00	0.00	0.00	0.00	0.00	0.00	0.01	0.00	0.00	0.00	0.96	0.00	0.00	0.00	0.00	0.02	0.01	0.00
	12	0.00	0.00	0.00	0.05	0.00	0.04	0.00	0.00	0.00	0.00	0.00	0.90	0.00	0.00	0.00	0.00	0.00	0.00
	13	0.00	0.00	0.00	0.01	0.12	0.00	0.00	0.00	0.00	0.01	0.00	0.00	0.86	0.00	0.00	0.00	0.00	0.00
	14	0.00	0.00	0.00	0.00	0.03	0.00	0.00	0.00	0.00	0.00	0.00	0.00	0.00	0.97	0.00	0.00	0.00	0.00
	15	0.00	0.00	0.00	0.00	0.00	0.00	0.00	0.00	0.00	0.00	0.00	0.00	0.00	0.00	1.00	0.00	0.00	0.00
	16	0.00	0.00	0.00	0.00	0.00	0.00	0.00	0.00	0.00	0.00	0.00	0.00	0.00	0.00	0.00	1.00	0.00	0.00
	17	0.00	0.01	0.00	0.00	0.00	0.00	0.00	0.00	0.00	0.00	0.01	0.00	0.00	0.00	0.00	0.00	0.98	0.00
	18	0.00	0.00	0.00	0.00	0.00	0.00	0.00	0.00	0.00	0.00	0.00	0.00	0.00	0.00	0.00	0.00	0.00	1.00
The combined biometrics		**1**	**2**	**3**	**4**	**5**	**6**	**7**	**8**	**9**	**10**	**11**	**12**	**13**	**14**	**15**	**16**	**17**	**18**
	1	1.00	0.00	0.00	0.00	0.00	0.00	0.00	0.00	0.00	0.00	0.00	0.00	0.00	0.00	0.00	0.00	0.00	0.00
	2	0.00	0.99	0.00	0.00	0.00	0.00	0.00	0.00	0.00	0.00	0.00	0.00	0.00	0.00	0.00	0.01	0.00	0.00
	3	0.00	0.00	0.99	0.00	0.00	0.01	0.00	0.00	0.00	0.00	0.00	0.00	0.00	0.00	0.00	0.00	0.00	0.00
	4	0.00	0.00	0.00	1.00	0.00	0.00	0.00	0.00	0.00	0.00	0.00	0.00	0.00	0.00	0.00	0.00	0.00	0.00
	5	0.00	0.00	0.00	0.00	1.00	0.00	0.00	0.00	0.00	0.00	0.00	0.00	0.00	0.00	0.00	0.00	0.00	0.00
	6	0.00	0.00	0.00	0.01	0.00	0.98	0.00	0.00	0.00	0.00	0.00	0.00	0.00	0.00	0.00	0.01	0.00	0.00
	7	0.00	0.00	0.00	0.00	0.00	0.00	1.00	0.00	0.00	0.00	0.00	0.00	0.00	0.00	0.00	0.00	0.00	0.00
	8	0.00	0.00	0.00	0.00	0.05	0.00	0.00	0.91	0.00	0.00	0.00	0.00	0.01	0.00	0.00	0.01	0.00	0.01
	9	0.00	0.00	0.00	0.00	0.00	0.00	0.00	0.02	0.93	0.01	0.00	0.00	0.02	0.01	0.00	0.00	0.00	0.00
	10	0.00	0.00	0.00	0.00	0.01	0.00	0.00	0.01	0.00	0.97	0.00	0.00	0.01	0.00	0.00	0.00	0.00	0.00
	11	0.00	0.00	0.00	0.00	0.00	0.00	0.00	0.00	0.00	0.00	0.98	0.00	0.00	0.00	0.00	0.01	0.01	0.00
	12	0.00	0.01	0.00	0.00	0.00	0.00	0.00	0.00	0.00	0.00	0.00	0.99	0.00	0.00	0.00	0.00	0.00	0.00
	13	0.00	0.00	0.00	0.00	0.02	0.00	0.00	0.00	0.00	0.00	0.00	0.00	0.98	0.00	0.00	0.00	0.00	0.00
	14	0.00	0.00	0.00	0.00	0.02	0.00	0.00	0.00	0.00	0.00	0.00	0.00	0.00	0.98	0.00	0.00	0.00	0.00
	15	0.00	0.00	0.00	0.00	0.00	0.01	0.00	0.00	0.00	0.00	0.00	0.00	0.00	0.00	0.99	0.00	0.00	0.00
	16	0.00	0.00	0.00	0.00	0.00	0.00	0.00	0.00	0.00	0.00	0.00	0.00	0.00	0.00	0.01	0.99	0.00	0.00
	17	0.00	0.01	0.00	0.00	0.00	0.00	0.00	0.00	0.00	0.00	0.00	0.00	0.00	0.00	0.00	0.00	0.99	0.00
	18	0.01	0.00	0.00	0.00	0.00	0.00	0.00	0.00	0.00	0.00	0.00	0.00	0.00	0.00	0.00	0.00	0.00	0.99

**Table 6:** Summary of the achieved identification accuracy results compared to literature.

Name	Biometric Identifier	Deep Model	No. of Participants	Accuracy
This work	Heart sound	GoogLeNet	18	58.7%
	Gait			96.2%
	Gait + Heart sound			98.0%
[2]	Heart µDoppler	CNN	10	80%
[4]	Gait	CNN	15	95.20%
[15]	Gait	LSTM-RNN	29	89.10%
[16]	Gait	TCN	10–100	97–89%
[17]	Gait	CNN	20	96.70%
[29]	Gait	TCN	5	94.90%
[30]	Gait	RAN-CNN	6	96.20%
[31]	Gait	ResNet-50	22	84%
[32]	Gait	CNN	29	86.9%,
[33]	Gait	AlexNet	4–20	97–69%

**Table 7:** The eight-subject confusion matrix for the practical testing results.

	1	2	3	4	5	6	7	8
1	0.9	0	0	0	0	0	0	0.1
2	0	0.4	0.2	0	0	0.1	0.2	0.1
3	0	0	0.9	0	0	0	0.1	0
4	0	0	0	0.9	0	0	0	0.1
5	0	0.2	0	0.1	0.4	0	0.3	0
6	0	0.1	0	0	0	0.9	0	0
7	0	0	0.2	0.1	0	0	0.7	0
8	0	0	0	0	0	0	0	1

## Data Availability

This study did not report any open-source data.
